# Case of Aneurism

**Published:** 1859-08

**Authors:** H. A. Spencer

**Affiliations:** Waterford, Pa.


WATERFORD, PA., May 16, 1859.

PROF. FLINT,—

*My Dear Sir*,—I send you the following case, which, if you think worthy your attention, you are at liberty to publish.

Some two months since, a son of Mr. T——, living eight miles from this place, aged 14 years, accidentally ran a knife-blade into the left thigh, unfortunately wounding the femoral artery, a little over two inches below where the profunda is given off. Strange as it may appear, the hemorrhage was very slight, and was easily suppressed by simple bandaging. This was the treatment that the case had until last Tuesday, the 10th inst., when I was called to see it. I found large false aneurism, and could distinguish the pulsation very easily. The wound made by the knife was partially closed; but upon my making a little exertion to introduce a small probe, a jet of blood passed by my head, going ten feet or more, and striking the chamber floor. I immediately checked the flow of blood, and made arrangements for cutting down and applying a ligature to the artery. I made an incision four or five inches in length, and found that the sac occupied about five inches of the artery. The whole cellular tissue around the sac was filled with coagulated blood, and the sac itself contained at least one pint. The sac reached almost to the profunda. I applied my ligature immediately below where that artery is given off. I then found that I had profuse hemorrhage from below by anastomosis, and immediately made my incision longer, followed down before the sac, and applied a ligature there, when all hemorrhage ceased at once. What made this operation very difficult, was the swelling being such that the incision had to be very deep, and the sac occupying so much of the artery, that in order to get above it, the ligature had to be applied very high up. These difficulties, taken with the length of time that elapsed after the injury before the operation was performed, and the great amount of blood thrown into the surrounding tissue, made the case a very perplexing one.

One week has now elapsed since the operation, and the young man is doing finely, and will make a perfect recovery. Dr. Humphrey assisted in the operation. I did not give anæsthetic; and the young man bore it like a hero. Yours, &c.,

H. A. SPENCER.

